# Intensity Simulation of a Fourier Transform Infrared Spectrometer

**DOI:** 10.3390/s20071833

**Published:** 2020-03-26

**Authors:** Zhuoya Ni, Qifeng Lu, Yishu Xu, Hongyuan Huo

**Affiliations:** 1 Key Laboratory of Radiometric Calibration and Validation for Environment Satellites, National Satellite Meteorological Center, China Meteorological Administration, Beijing 100081, China; nizy@cma.gov.cn; 2 State Key Laboratory of Severe Weather, Chinese Academy of Meteorological Sciences, Beijing 100081, China; 3 Chinese Academy of Meteorological Sciences, Beijing 100081, China; xuyishusuzhou@126.com; 4 College of Architecture and Civil Engineering, Beijing University of Technology, Beijing 100124, China; hongyuanh@gmail.com

**Keywords:** Michelson interferometer, FY-4B GIIRS, line shape function, off-axis effects, non-linearity, Fourier transform spectrometer

## Abstract

This paper introduces an intensity simulation for the Fourier transform infrared spectrometer whose core element is the Michelson interferometer to provide support for the on-orbit monitoring of the instrument and to improve the data processing and application of the Fourier transform spectrometer. The Geostationary Interferometric Infrared Imager (GIIRS) aboard on Fengyun-4B (FY-4B) satellite, which will be launched in 2020, aims to provide hyperspectral infrared observations. An intensity simulation of the Michelson interferometer based on the GIIRS’s instrument parameters is systematically analyzed in this paper. Off-axis effects and non-linearity response are two important factors to be considered in this simulation. Off-axis effects mainly cause the wavenumber shift to induce a large brightness temperature error compared with the input spectrum, and the non-linearity response reduces the energy received by the detector. Then, off-axis effects and a non-linearity response are added to the input spectrum successively to obtain the final spectrum. Off-axis correction and non-linearity correction are also developed to give a full simulation process. Comparing the corrected spectrum with the input spectrum, we can see that the brightness temperature errors have a magnitude of 10^−3^ K, and this fully proves the reliability and rationality of the whole simulation process.

## 1. Introduction

The Michelson interferometer is a precision optical instrument designed and manufactured by American physicist Michelson and Morey in 1883, with the aim of studying the “ether” drift. It is a split-amplitude interferometer, which generates interference by splitting the amplitude of the input light into two parts, and by adjusting the distance of the moving mirror from the beam splitter, changes in interference fringes can be observed [1,2]. Rayleigh found that the interferogram generated from the Michelson interferometer has a mathematical relationship with the spectrum of the light source, and he obtained the spectrum using the Fourier transform [3]. Under this principle, a series of spectrometer based on the Michelson interferometer have been designed, such as the Infrared Atmospheric Sounding Interferometer (IASI) carried on the MetOp satellite [4,5,6], the Cross-Track Infrared Sounder (CrIS) carried aboard the Suomi National Polar-Orbiting Partnership (NPP) satellite [7,8,9], the High-Spectral Resolution Infrared Atmospheric Sounder (HIRAS) aboard on FY-3D satellite [10,11,12], and the Geostationary Interferometric Infrared Imager (GIIRS) aboard on the FY-4A satellite [13,14]. Interferometric spectroscopy technology, taking the advantages of high luminous flux and multi-channels, has been developed in many fields, such as military reconnaissance [15,16], astrophysics research [17], geological resource exploration [18], and atmospheric monitoring [19,20,21,22,23,24,25]. Interferogram is not only the direct result of the Michelson interferometer, but it is also the basis for obtaining the spectrum of the light source.

FY-4A GIIRS is the first space-borne interferometer in geostationary orbit, and it measures the three-dimensional atmospheric structure through the interference of split light beams. Based on the FY-4A’s success, the FY-4B satellite will be launched in 2020. The GIIRS will also the main important payload instrument of the FY-4B satellite. Research regarding the intensity simulation of the GIIRS has been carried out to analyze the effects of the instrument’s parameters, diagnose and optimize the instrument’s on-orbit parameters by analyzing the relationship between simulation status and instrument on-orbit parameters and track and monitor instrument status and data quality.

Circular detectors and square detectors are commonly used in flat array detectors: the CrIS focal plane has nine circular detectors arranged as a 3 × 3 array [7,26]; the FY-4A GIIRS focal plane has 128 square detectors arranged as a 32 × 4 array [14,27]; and the FY-4B GIIRS focal plane has 128 square detectors arranged as a 16 × 8 array. The finite aperture of the interferometer, an off-axis detector, and other factors can cause off-axis effects. Off-axis effects mainly result in a wavenumber shift. Therefore, off-axis effects are the important factor in this simulation. Further, detector non-linearity can introduce radiometric errors and reduce the energy received by the detector, and it should be corrected to reduce the radiometric error. In this paper, these two factors are considered and analyzed in the simulation process.

This paper demonstrates an interference intensity simulation of the Michelson interferometer following the FY-4B GIIRS’s instrument parameters, including off-axis effects and the nonlinearity response. In this article, several parts of the simulation process are connected to demonstrate completely the intensity simulation from theory to practice. The theory regarding off-axis effects and the nonlinearity response are introduced first, including the reasons these effects exist, the computation strategies, and the calibration methods. Then, simulation data including off-axis effects or the non-linearity response are generated to assess the effects of these factors. Finally, these two factors are combined to be added to the input spectrum to obtain the final intensity simulation data. To prove the reliability of the whole simulation process, corresponding calibration processes are also developed. In this paper, a complete and detailed intensity simulation process, including off-axis effects and the non-linearity response, are shown and analyzed, and this will provide the support for subsequent on-orbit testing.

## 2. The Introduction of the Michelson Interferometer

### 2.1. The Principle of the Michelson Interferometer

**Figure 1:**
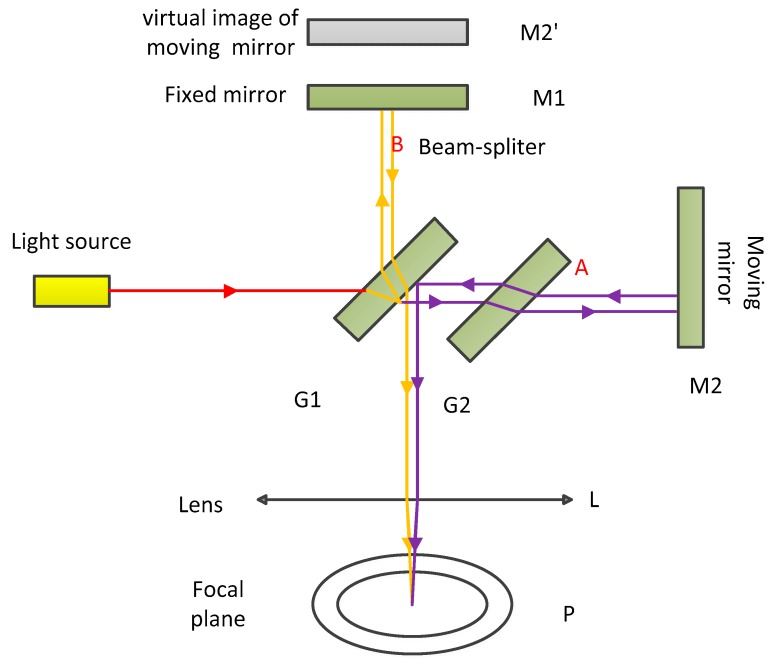
Schematic diagram of the Michelson interferometer.

Figure 1 depicts the schematic of the Michelson interferometer [28]. In the Michelson interferometer, the direction perpendicular to the moving mirror is defined as the on-axis. The light source, a beam of light along the direction of the on-axis, passes through a beam splitter (the glass is coated with a translucent silver film so that half of the incident light is refracted and half is reflected) so that it is split into two beams, A and B, before being emitted back through two mirrors. Interference fringes are formed after their meeting. One of the two mirrors can be moved, referred to as the moving mirror. Its movement will cause a change in the optical path difference of this beam, resulting in a change of the interference fringes. Additionally, the moving distance of the moving mirror is related to the number of changes in the interference fringes. M2’ is the virtual image of the M2 by the beam splitter G1. In other words, the optical interference here is equivalent to the air plate interference between M2’ and M1. The role of the Michelson interferometer is to create such a layer of imaginary air. The reason for the setting compensation plate G2 is to compensate for the dispersion of G1 when a white light source is used.

Suppose that monochromatic light with an incident wavelength of λ and amplitude E_0_, enters into the interferometer, the total intensity in the focal plane is expressed as:

(1) $$I_{total}=B(1+\cos(2\pi x/\lambda ))=B(1+\cos(2\pi \sigma x))$$

in which, *B* = 2(0.5 × E_0_)^2^, and *x* is the optical path difference.

In the interference of an ideal point light source, the incident light is monochromatic. In fact, the light source is not an ideal point light source but a surface light source. When the surface light source enters the Michelson interferometer, light waves that have the same frequency will interfere, and the total energy recorded by the Michelson interferometer will be the superposition of the interference light energy of the monochromatic light of different frequencies, which can be expressed as:

(2) $$I_{total}\;=\int_{\sigma_{1}}^{\sigma_{2}}B(1+\cos(2\pi \sigma x))d\sigma$$

### 2.2. The Off-Axis Effects of the Flat Array Detector

In the case of a point light source, the light travels along the optical axis and reaches the center of the focal plane. In fact, to detect the signal, the light source is a general surface light source or extended light source. In the case of the extended light source, the incident rays have a certain field of view, and some incident rays travel in the Michelson interferometer along the direction of the off-axis. The extended light source is a factor in determining the instrumental line shape (ILS) [29]. The ILS represents the contribution of instrumental finite resolution and all other contributors such as instrument misalignments and shear [7]. In other words, the ILS can be thought of as the response of the monochromatic light to the instrument and is affected by such things as the truncation, finite field of view, off-axis, and diffraction effects of the Fourier transform spectrometer [30,31].

**Figure 2:**
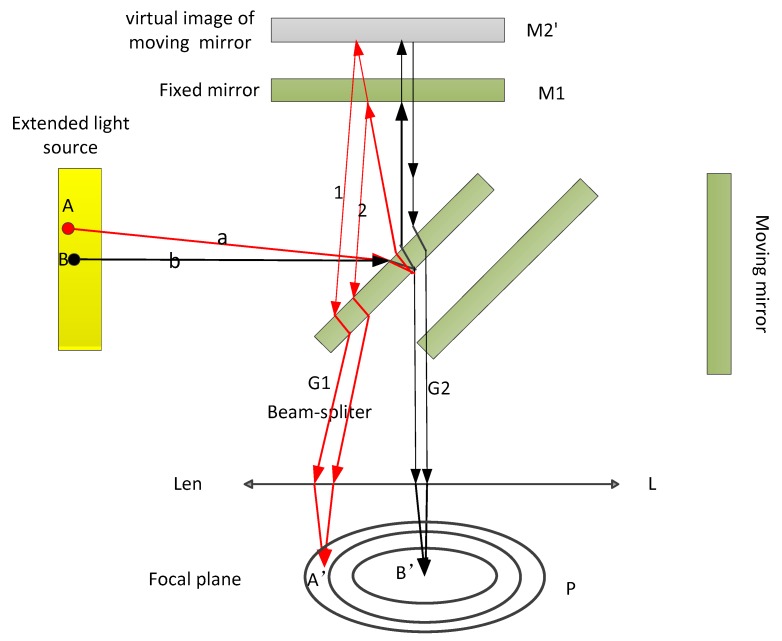
Off-axis light in the Michelson interferometer. The light wave passes through the mirror and lens, and arrives at the focal plane. Light sources A and B represent the different positions of the extended light source.

As shown in Figure 2, the light source is the extended light source. The direction perpendicular to the moving mirror or moving mirror virtual image is defined as the on-axis (e.g., direction b); the incident light in this direction is reflected by the beam splitter and then reflected by the fixed mirror and the moving mirror virtual image. The two rays along the light direction of the center of the convergence lens reach focus point B’ in the focal plane. In the case of an extended light source, the rays along direction ‘a’ reach the beam splitter mirror and are reflected by the beam splitter mirror to the fixed mirror and the virtual image of the moving mirror, then the rays are reflected by the fixed mirror and the moving mirror virtual image, and thus rays 1 and light 2 are formed. Rays 1 and 2 are parallel coherent lights with certain optical path differences. The two beams of coherent light pass through the convergence lens and reach the focal plane A’ point. The angle between rays 1 and 2 and the on-axis b is θ, and the angle θ can be called the off-axis angle.

Assuming that the optical path difference on-axis is *x*, the light interference intensity at the focus of the focal plane can be expressed as

(3) I =Bcos(2πσx)

**Figure 3:**
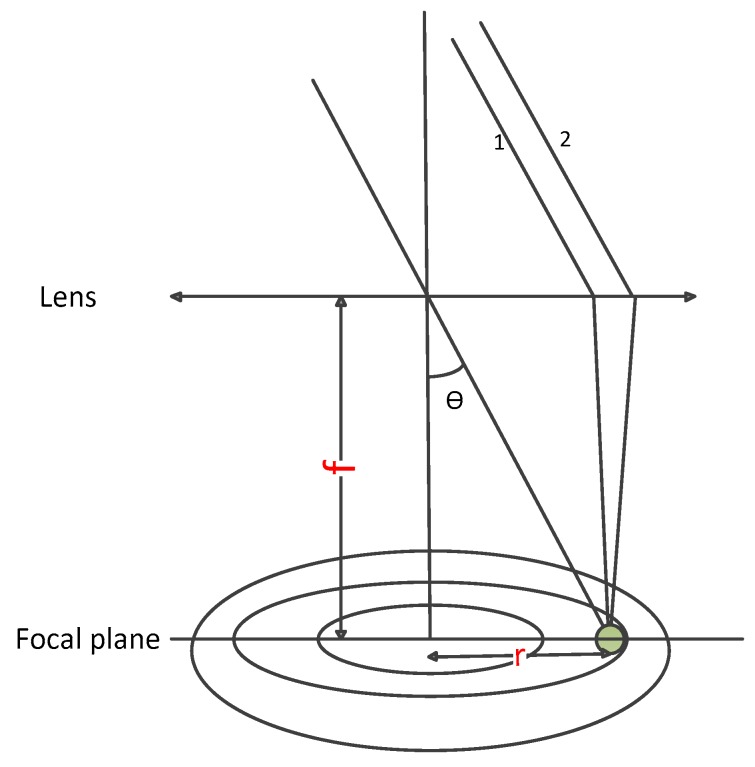
Geometry of the detector for the off-axis position.

In Figure 3, the off-axis angle is *θ*, and the intensity of light recorded on the focal plane is

(4) I =B·cos(2πσx·cosθ)

Equation (5) can be regarded as the case where the optical path difference is constant and the wave number is expanded:

(5) I =B·cos(2πσ·cosθ·x)

The array of detectors on the focal plane is generally circular or square. In the case of the GIIRS, the detector is square. Hence, square detectors at different positions on the focal plane are taken as an example to analyze the variation of the ILS of the Michelson interferometer due to off-axis effects.

According to the imaging principle of the convex lens, the light parallel to rays 1 and 2 passes through the direction of the centroid of the condenser lens, and is the same as the position where rays 1 and 2 are concentrated by the lens (Figure 3). ***r*** is defined as the distance from the detector to the center of the focal plane, and the geometric relationship can be obtained by

(6) r=f·tan(θ)

In Equation (5), the new variable *σ_n_* expressed by the actual wave number and off-axis angle, is used to express the apparent wave number:

(7) $$\sigma_{n}=\sigma \cdot \cos\theta$$

When Equation (6) is combined with Equation (7), the other expression of ***r*** can be derived:

(8) $$r=f\cdot \sqrt{\sigma^{2}/\sigma_{n}{}^{2}-1}$$

## 3. The Theoretical Framework of the Intensity Simulation of the GIIRS

**Figure 4:**
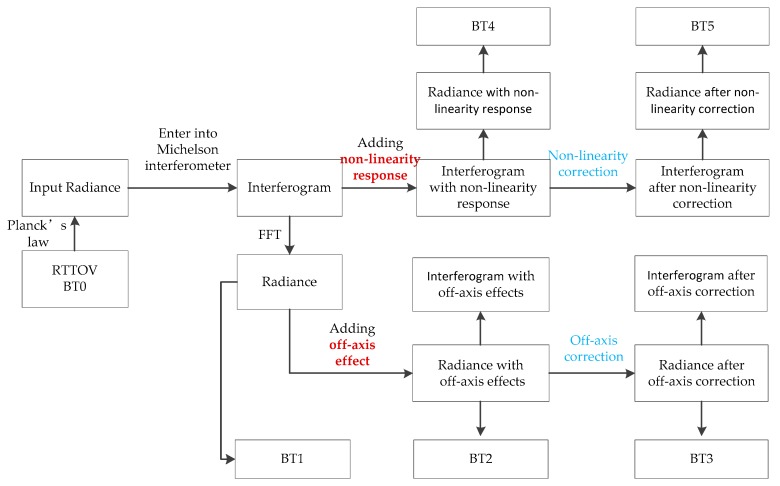
Flow chart of the analysis of off-axis effects and the non-linearity response.

**Figure 5:**
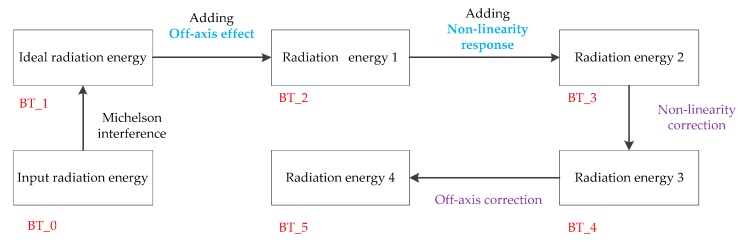
Flow chart of simulation process.

The aim of this paper is to develop an intensity simulation of the GIIRS, including the off-axis effect and nonlinear response. In the first, the off-axis effects and non-linear response are analyzed respectively (See Figure 4), and then the intensity simulation, including off-axis effects and the non-linear response is developed (see Figure 5). Because the interferogram is more abstract, the radiance and brightness temperature (BT) are used to compare the changes before and after each operation using the characteristic values.

In Figure 4, the off-axis effects and non-linearity response are analyzed separately. In Figure 5, the full simulation process including off-axis effects and the non-linearity response, is depicted. Following the order of adding the off-axis effect, non-linear response, non-linearity correction and off-axis correction, the changes in BT error are used to illustrate the reliability of the full simulation process.

### 3.1. Introduction of the Simulation Set

**Figure 6:**
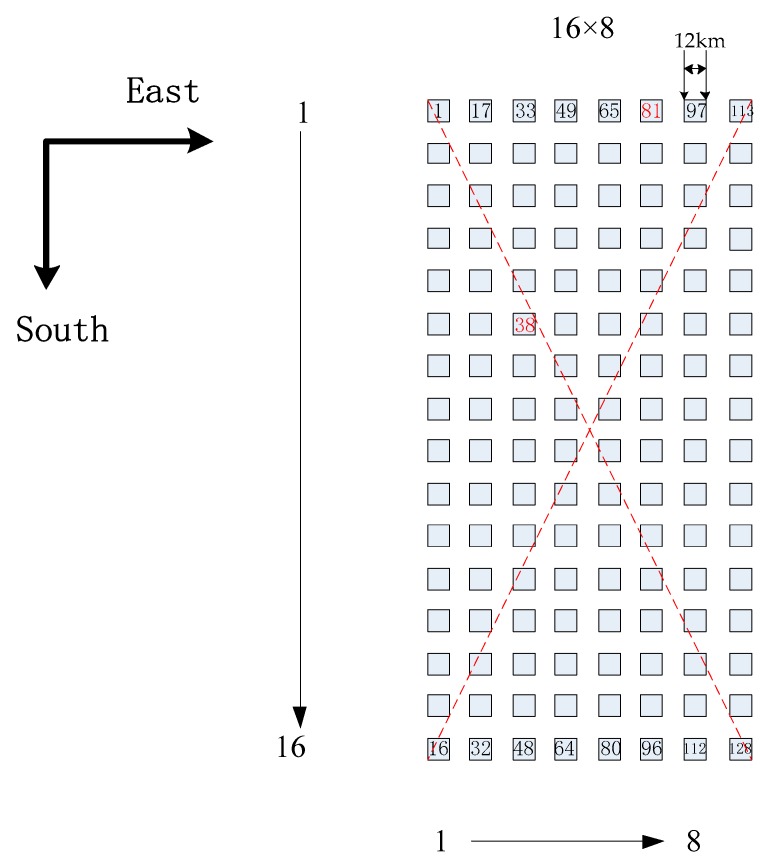
The placement of detectors in the focal plane of the Geostationary Interferometric Infrared Imager (GIIRS).

In this section, the Fourier transform spectrometer is taken as an example to show the results of the ILS and intensity computation. FY-4B GIIRS is the space-borne interferometer in geostationary orbit, which we use to measure the three-dimensional atmospheric structure, whose core instrument is a Michelson interferometer. In this simulation, the input spectral bands are medium wave (680–1130 cm^−1^), and the placement of detectors in the focal plane of the GIIRS is given in Figure 6. Radiative Transfer for TOVS (RTTOV) is a fast radiative transfer model, which is widely used in the satellite retrieval and data assimilation communities [32], and is used to simulate the input brightness temperature using the GIIRS’s instrument parameters. In the Michelson interferometer, equal optical path difference sampling is generally used to control data acquisition using a He-Ne laser. If the reference laser wavelength is $\lambda_{\mathrm{laser}}$, and the optical path difference interval is the laser wavelength, then:

(9) $$\Delta x=\lambda_{laser}$$

### 3.2. Computation of the ILS

**Figure 7:**
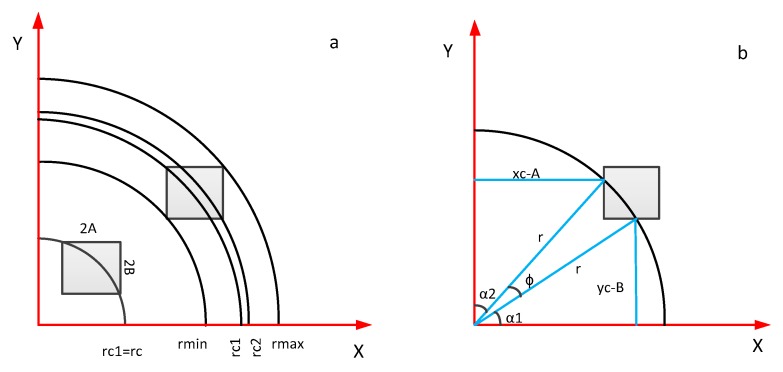
Geometry of a square detector for the off-axis positions in the focal plane: (**a**) different sites of the detector; (**b**) the arc segmented by the detector.

In Figure 7a, the ratio of the arc segmented by the square detector to the entire circle is the ILS function. Suppose the points on the detector are represented by the polar coordinates (r, φ), in which r is the distance between the detector and the optical axis, and φ is defined as the opening angle of the arc segmented by the detector, and the half width and half height of the square detector are A and B, respectively. The coordinates of the four corner points can be expressed as follows:

(10) $$\begin{array}{l} r_{\min}={\left({\left(x_{c}-A\right)}^{2}+{\left(y_{c}-B\right)}^{2}\right)}^{1/2}\\ r_{c1}={\left({\left(x_{c}-A\right)}^{2}+{\left(y_{c}+B\right)}^{2}\right)}^{1/2}\\ r_{c2}={\left({\left(x_{c}+A\right)}^{2}+{\left(y_{c}-B\right)}^{2}\right)}^{1/2}\\ r_{\max}={\left({\left(x_{c}+A\right)}^{2}+{\left(y_{c}+B\right)}^{2}\right)}^{1/2} \end{array}$$

As shown in Figure 7b, the arc segmented by the rectangular detector occupies the proportion of the entire ring; that is, the normalized arc segment can be expressed as follows:

(11) $$I_{n}=\frac{1}{2\pi }(\frac{\pi }{2}-\alpha_{1}-\alpha_{2})$$

in which, $\alpha_{1}$ and $\alpha_{2}$ are opening angles of different arcs. In Figure 7b, the rectangular detector is divided into three regions by circles of different radii, and the expression of the ILS function is synthesized using these three arcs. One should pay attention to distinguishing the sizes of rc1 and rc2 because this decides the different expression of the ILS function. Based on the above analysis, the expressions of the ILS function in different situations are given in Table A1 [33].

### 3.3. Intensity Computation of the Detector for the Off-Axis Position

**Figure 8:**
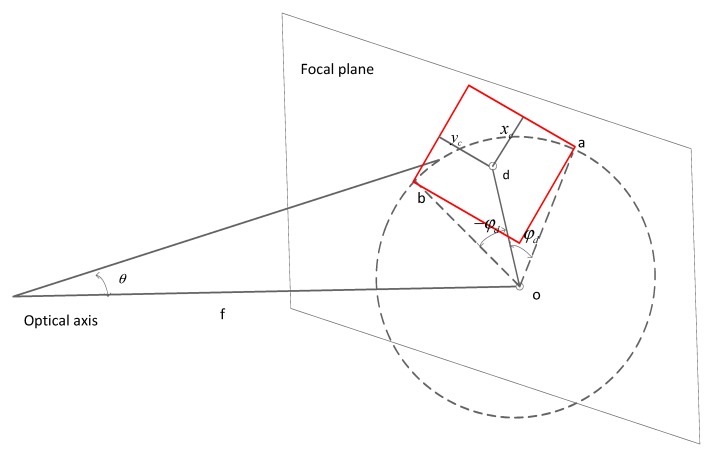
A light ray with an off-axis angle θ is intercepted by the square detector, whose half-width and half-height are xc and yc, respectively. In the focal plane, rays in the dash circle have the same off-axis angle, and the opening angle of the arc intercepted by the detector is in the range of $-\varphi_{d}$ and $\varphi_{d}$. “f” is the focal length.

In terms of the extended light source, the total radiation energy received by the detector is the integration of the rays over the solid angle $\Omega_{d}$. If only considering the intensity received by the detector at angle *θ* within the solid angle $\Omega_{d}$ (See Figure 8), the equation is written as follows:

(12) $$d^{2}I=\;\int_{\upsilon }Bcos(2\pi \sigma x\cdot cos\theta )d\upsilon d\Omega /\Omega_{d}$$

The total radiance energy received by the detector is computed by integral form at the solid angle range corresponding to the entire detector. The calculation formula is expressed as follows [7]:

(13) $$I=\int_{\Omega }\int_{\sigma }Bcos(2\pi \sigma x\cdot cos\theta )d\sigma d\Omega /\Omega_{d}$$

Based on the characteristics of the small solid angle, *d*Ω can be expressed as sin*θdθdφ*. *θ* is the off-axis angle, and *φ* is the opening angle of the ring on the focal plane. Equation (13) can be written as follows:

(14) $$\begin{array}{l} I=\int_{\theta }\int_{-\varphi_{d}}^{\varphi_{d}}\sin\theta \int_{\upsilon }B(\sigma )\cdot cos(2\pi \sigma x\cdot cos\theta )d\sigma d\theta d\varphi /\Omega_{d}\\ \;\;\;\;\;\;\;=-\int_{\alpha }\int_{\sigma }B(\sigma )\cdot cos(2\pi \sigma x\cdot \alpha )d\sigma \cdot 2\varphi_{d}/\Omega_{d}d\alpha \\ \;\;\;\;\;\;\;=-\int_{\alpha }\int_{\sigma }B(\sigma )\cdot cos(2\pi \sigma x\cdot \alpha )\cdot ILS(\alpha )d\sigma \cdot d\alpha \\ \;\;\;\;\;\;\;=-\int_{\sigma_{n}}(\int_{\sigma }B(\sigma )\cdot ILS(\sigma_{n}/\sigma )d\sigma )\cdot cos(2\pi \sigma_{n}x)\cdot 1/\sigma d\sigma_{n}\\ \;\;\;\;\;\;\;=-\int_{\sigma_{n}}(\int_{\sigma }B(\sigma )\cdot ILS(\sigma_{n}/\sigma )\cdot 1/\sigma d\sigma )\cdot cos(2\pi \sigma_{n}x)d\sigma_{n} \end{array}$$

in which $\alpha =\cos(\theta )=\sigma_{n}/\sigma$.

### 3.4. Nonlinearity Response of the Detector

In general, the output signal of the detector has a linear relationship with the input radiation energy. If the radiation energy exceeds a certain value, the relationship between the output signal and input radiation, energy becomes non-linear. The interference intensity includes the DC part (B) and AC part (I), subscript m indicates the actual interference signal, and subscript i indicates the ideal signal. Supposing that the ideal signal has a quadratic linear relationship with the actual signal, the expression is as follows:

(15) $$I_{i}+B_{i}=I_{m}+B_{m}+a_{2}{\left(I_{m}+B_{m}\right)}^{2}$$

Equation (15) can be written in other forms:

(16) $$I_{m}+B_{m}=I_{i}+B_{i}-a_{2}{\left(I_{m}+B_{m}\right)}^{2}$$

In the simulation process, the ideal intensity and the initial parameter $a_{2}$ are known parameters, and the actual intensity with the nonlinear response is a required parameter. Suppose the quadratic square of the actual signal is close to the quadratic square of the ideal signal, then Equation (16) is rewritten as follows:

(17) $$I_{m}+B_{m}=I_{i}+B_{i}-a_{2}{\left(I_{i}+B_{i}\right)}^{2}$$

Expanding Equation (17) and eliminating the DC terms produces the following:

(18) $$I_{m}=I_{i}-2a_{2}I_{i}B_{i}-a_{2}I_{i}{}^{2}$$

Through Equation (18), the actual output signal with non-linearity distortion is obtained. In Equation (15), the DC part has no effect on the final results. By eliminating the DC part and transforming the spectral domain, Equation (15) can be expressed as Equation (19). Many researchers have shown that in-band distortion is caused by the first term on the right of Equation (19), and out-of-band distortion is caused by the second term [7,34]. In-band distortion and out-of-band distortion are independent from each other. The out-of-band spectrum is used to compute $a_{2}$.

(19) $$Spec_{i}=(1+2a_{2}B_{m})Spec_{m}+a_{2}Spec_{m}⊗Spec_{m}$$

In ideal conditions, in the low-frequency out-of-band region, the ideal interferogram is zero. Equation (19) can be rewritten in other forms:

(20) $$Spec_{m}+2a_{2}B_{m}Spec_{m}+a_{2}Spec_{m}⊗Spec_{m}=0$$

In Equation (20), $Spec_{m}$ and *B_m_* are known parameters, and $a_{2}$. can be computed through this method in the low frequency out-of-band region. Following the parameters set for the CrIS non-linearity correction, the low-frequency bands are also set from 50 to 300 cm^−1^, and this means that the parameters in Equation (20) are set from 50 to 300 cm^−1^.

(21) $$a_{2}=a_{2}{}^{\prime }/(1-2B_{m}a_{2}{}^{\prime }),\;a_{2}{}^{\prime }=-Spec_{m}/(Spec_{m}⊗Spec_{m})$$

If parameter $a_{2}$ is obtained, the measured spectrum including non-linearity can be corrected by Equation (22). The explanation of the parameters are stated in the Table 1.

(22) $$Spec_{i}=(1+2a_{2}V_{m})Spec_{m}$$

## 4. Analysis of the Simulation Results of the Fourier Transform Spectrometer GIIRS

### 4.1. ILS for Various Positions of Detectors

Off-axis effects are generated by the finite aperture of the interferometer and other optical, mechanical and electrical imperfections of the interferometer system. Both the detector size and its positions on the focal plane define the width and shape of the spectral lines [7]. When the light source has a limited field, two-beam monochromatic light with a certain optical difference passes through the lens and generates the interference fringes on the focal plane. The ILS is defined as the shape change of the monochromatic light passing through the instrument to the focal plane and is affected by the limited field, off-axis effect, the limited moving range of the moving mirror, optical diffraction and moving mirror misalignment. In this work, the Michelson interferometer is considered the ideal condition, and the limited field and off-axis effect are considered the main factors affecting the ILS.

**Figure 9:**
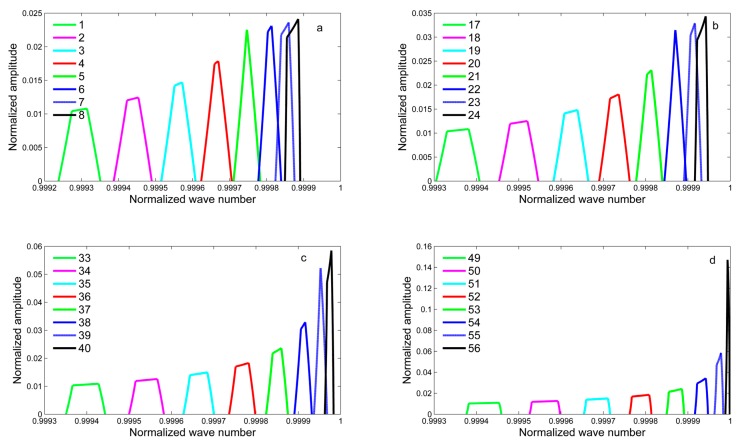
The instrumental line shape (ILS) of typical detectors in the focal plane of GIIRS.

There are 128 detectors on the focal plane, and there are 128 line shapes due to their different positions. In the case ofFY-4B GIIRS, no detector is in the on-axis position, and all detectors are in off-axis positions. Because of the symmetry of the focal plane, the detectors in the left half of the focal plane are selected to illustrate the variation of the ILS. Following the ILS computation of a square detector in Section 3.1, the ILS of the selected typical detectors are showed in the Figure 9. The horizontal axis is the normalized wavenumber (σ_n_/σ), and the vertical axis is the normalized arc length intercepted by the detector corresponding to different off-axis angles.

Comparing these detectors for different positions, we reach several conclusions:

(1) Detectors for the different positions have different ILS values. The variations of the positions cause that detectors intercept different arc lengths corresponding to the same off-axis angles. Compared with on-axis detector, off-axis detector, which is far from the central point of the FOV, intercepts the smaller arc length, which means that the ILS value is relatively small. This conclusion can be found in Figure 9. In other columns, from the bottom to up, the ILS values decrease gradually.

(2) Off-axis effects cause the changes of the width and shape of the ILS. The width of the spectral line widens, and spectral stretching causes the ILS to be shifted to a low wave number. From Figure 9, it is clear that the spectral stretching and shifting of detectors at both ends of the column for off-axis positions are much more serious than for on-axis detectors. It is necessary to remove off-axis effects in the process of correcting the instrument spectra.

Figure 9 gives the ILS functions for off-axis positions. In these conditions, the ILS function of a square detector for off-axis positions is thought of as the piecewise function. Attention should be paid to the relationship between rc1 and rc2. When rc1 equals rc2, the ILS functions include the contribution of two arcs; in contrast, ILS functions are a synthesis of three arcs with clear asymmetry. From Figure 9, it can be clearly seen that the variation of the ILS with the change between the detector and the optical axis and far from the optical axis causes the ILS function to shift to a low wavenumber. Therefore, correction of the spectral frequency in the stage of spectral calibration should be considered.

### 4.2. Intensity Analysis with Off-Axis Effects

**Figure 10:**
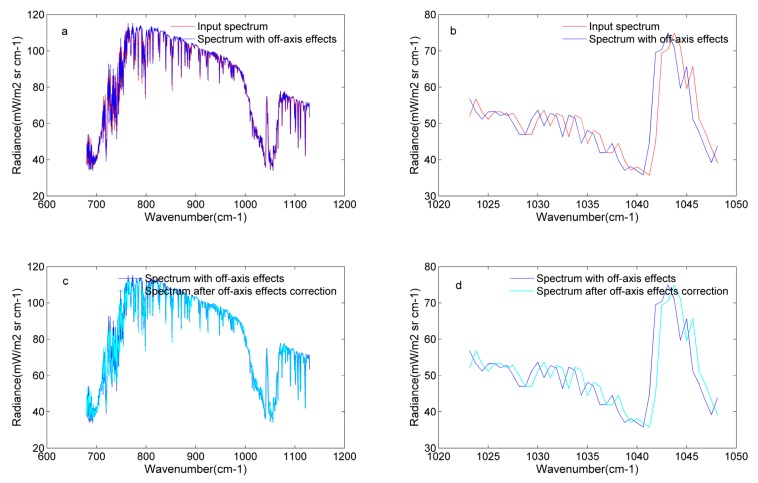
Analysis of off-axis effects for detector 81: (**a**) comparison between the input spectrum and the spectrum with off-axis effects from 680 to 1130 cm^−1^; (**b**) comparison between the input spectrum and the spectrum with off-axis effects from 1023 to 1048 cm^−1^; (**c**) comparison between the spectrum with off-axis effects and the spectrum after off-axis correction from 680 to 1130 cm^−1^; (**d**) comparison between the spectrum with off-axis effects and the spectrum after off-axis correction from 1023 to 1048 cm^−1^.

Based on the introduction of spectral ILS distortions due to an off-axis detector, Section 3.2 gave the interference intensity formula of a square detector for different positions, and the square detector is taken as an example to show the results of intensity simulation. Equation (12) shows that intensity values of every wavenumber are accumulated at the same optical path difference, and the final interferogram is the sum of the intensity of all wavenumbers at different optical path differences over the solid angle $\Omega_{d}$. The interferogram is relatively abstract and is difficult to understand. Therefore, the Fourier transform is applied to the interferogram to obtain its spectrum. Through comparing the radiance variation, the off-axis effects are illustrated in Figure 10.

In Figure 10, the off-axis effect of the GIIRS is computed and added to the input original spectrum. In this process, the off-axis effect is the only factor to affect the output spectrum. It should be noted that adding off-axis effects and off-axis correction is carried out in the spectrum domain (see Figure 4). Because of the finite optical path of the Michelson interferometer, the actual interferogram is truncated by the rectangular window function. The Fourier transform of the rectangular window function is the sinc function in the spectrum domain. This is called the self-apodization effect. The self-apodization effect and ILS function are also combined as a matrix, called the SA matrix. When adding off-axis effects, the original spectrum is multiplied by the SA matrix; when correcting off-axis effects, the spectrum with the off-axis effects is multiplied by the inverse of the SA matrix (SA^−1^).

Figure 10a,b gives the comparisons between the input spectrum (red line) and the spectrum with off-axis effects (blue line). It was found that the off-axis effects cause the spectrum to shift to a low wave number. Compared with the input radiance in the same wavenumber, the radiance with off-axis effects has changed. Figure 10c,d illustrates that off-axis correction can correct the distortions of the spectrum or interferogram. After off-axis correction, the correct wavenumbers are assigned to the affected spectrum according to the detectors’ positions. It also reveals that the wavenumber shift should be noted in the ground data processing system.

**Figure 11:**
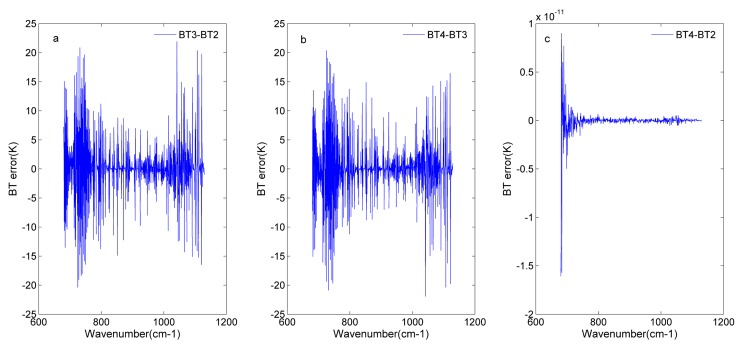
Brightness temperature (BT) error before and after off-axis correction for detector 81: (**a**) BT error in the input spectrum (BT2) and the spectrum with off-axis effects (BT3); (**b**) BT error in the spectrum with off-axis effects (BT3) and the spectrum after off-axis correction (BT4); (**c**) BT error in the spectrum after off-axis correction (BT4) and the input spectrum (BT2).

In Figure 11, the BT error is taken as the indicator to assess the off-axis effects and off-axis correction. Figure 11a illustrates that adding the off-axis effects introduces a large BT error to the original signal. Off-axis effects cause the wavenumber shift. In the same wavenumber, the radiance has a large difference due to many absorption lines. The BT is computed from the radiance through the Planck’s function. Therefore, the off-axis effects induce a large BT error (See Figure 11b). After off-axis correction, the BT error decrease to the magnitude of 10^−11^ K in Figure 11c. This also illustrates that the off-axis effects are effectively removed.

### 4.3. Analysis of the Nonlinearity Response

**Figure 12:**
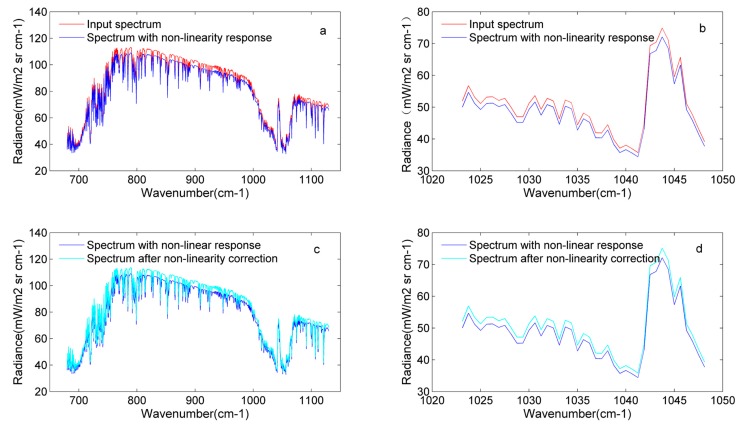
Analysis of the nonlinearity response for detector 81: (**a**) comparison between the input spectrum and the spectrum with a nonlinearity response from 680 to 1130 cm^−1^; (**b**) comparison between the input spectrum and the spectrum with the nonlinearity response from 1023 to 1048 cm^−1^; (**c**) comparison between the spectrum with the nonlinearity response and the spectrum after nonlinearity correction from 680 to 1130 cm^−1^; (**d**) comparison between the spectrum with the nonlinearity response and the spectrum after nonlinearity correction from 1023 to 1048 cm^−1^.

Similarly to the analysis of the off-axis effects, the non-linearity response is added to the input original interferogram, and the non-linearity correction for the in-band spectrum is carried out by estimating the correction parameter in the low wave number out-of-band spectrum. The non-linearity response can lose the energy received by the detector. In Figure 12a,b, it is clear that the radiance with the non-linearity response is less than the input radiance. Through the non-linearity correction, the lost energy is compensated for (See Figure 12c,d). The processing of the non-linearity response is developed in the time domain, which means that the operation occurs in the interferogram.

**Figure 13:**
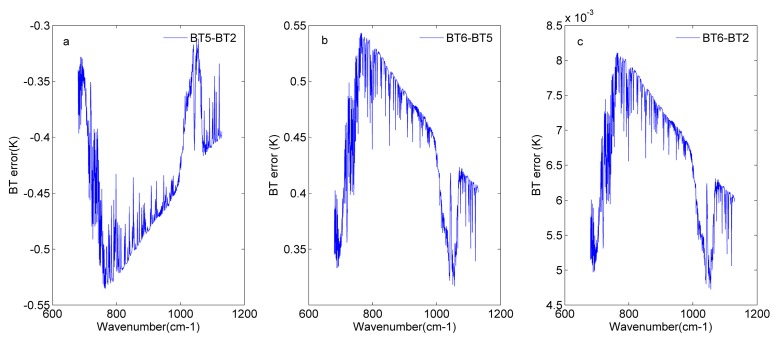
BT errors before and after nonlinearity correction for detector 81: (**a**) BT error in the input spectrum (BT2) and the spectrum with the nonlinearity response (BT5); (**b**) BT error in the spectrum with nonlinearity response (BT5) and the spectrum after nonlinearity correction (BT6); (**c**) BT error in the spectrum after nonlinearity correction (BT6) and the input spectrum (BT2).

The BT error is also used to illustrate the error introduced by the non-linearity response. Figure 13a shows that adding the non-linearity response introduces about 0.25 K BT errors. Figure 13b gives the comparison of BT errors before and after the non-linearity correction, and shows that non-linearity correction can compensate for the lost energy to avoid the energy loss received by the detector. To illustrate the rationality of the nonlinear correction process, the comparison between the input BT and BT after the non-linearity correction is demonstrated in Figure 11c. The result reveals that the BT error is 10^−3^ in magnitude and the whole process is reliable. Equation (22) shows that the parameter $a_{2}$ is important in the process of non-linearity correction. The estimated accuracy of $a_{2}$ can affect the results of non-linearity correction.

### 4.4. Intensity Simulation with Off-Axis Effects and the Non-Linearity Response

In Section 4.3 and Section 4.4, the off-axis effects and non-linearity response were analyzed, respectively. In this section, the off-axis effects are added to the input spectrum to obtain the new spectrum, and then the non-linearity response is added to the new spectrum. Based on the GIIRS’s instrument parameters, a spectrum including the off-axis effects and non-linearity response is obtained. To prove the reliability of the simulation process, the nonlinearity correction and off-axis correction are applied to the simulated spectrum. In this paper, the BT error is used as the indicator to illustrate the reliability of the simulation process. In the focal plane of the GIIRS, there are 128 detectors. The simulated results of two detectors are shown in this section.

**Figure 14:**
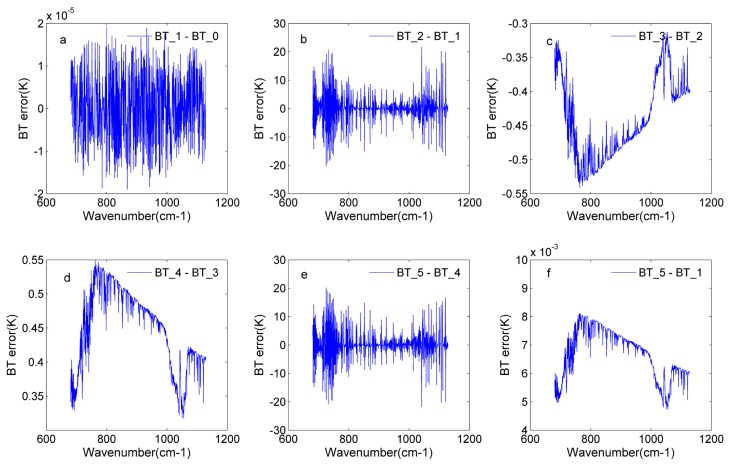
The BT error for detector 81 in the whole simulation process: (**a**) the BT error in the input BT (BT_0) and ideal BT (BT_1); (**b**) the BT error in the BT with off-axis effects (BT_2) and the ideal BT; (**c**) the BT error in the BT with off-axis effects and non-linearity response (BT_3) and BT_2; (**d**) the BT error in the BT after non-linearity correction (BT_4) and BT_3; (**e**) the BT error in the BT after off-axis correction (BT_5) and BT_4; f) the BT error in the BT_5 and BT_1.

**Figure 15:**
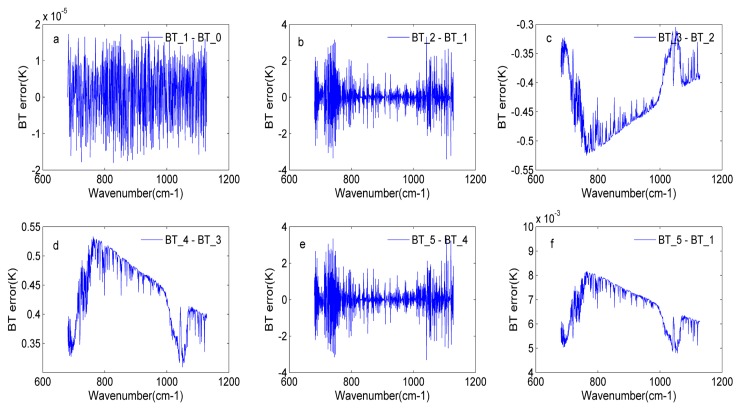
The BT error for detector 38 in the whole simulation process: (**a**) the BT error in the input BT (BT_0) and ideal BT (BT_1); (**b**) the BT error in the BT with off-axis effects (BT_2) and the ideal BT; (**c**) the BT error in the BT with off-axis effects and non-linearity response (BT_3) and BT_2; (**d**) the BT error in the BT after non-linearity correction (BT_4) and BT_3; (**e**) the BT error between BT after off-axis correction (BT_5) and BT_4; (**f**) the BT error in BT_5 and BT_1.

Figure 14 shows the simulated results of detector 81. The input BT_0 is simulated by RTTOV. The input radiance is resampled by the instrument following the reference laser wavelength, and changes to the ideal radiance interacting with the instrument. Based on the ideal radiance, the off-axis effects and non-linearity response are added successively, and the results of each step are expressed using the BT_, namely BT_2 and BT_3. To give a complete simulate process, the off-axis correction and non-linear correction are carried out based on the simulation data. It should be noted that the non-linearity correction is performed first in this paper. The results of the non-linearity correction and off-effects correction are expressed as BT_4 and BT_5. The result of the current operation is compared with the result of the previous step to show the introduced error by the current operation. Figure 14a illustrates that the difference between the input BT and the ideal BT is in the range of −1.5 × 10^−5^ K and 2 × 10^−5^ K. It reveals that the energy loss can be neglected in this operation. Figure 12b introduces the BT error brought by the off-axis effects. For detector 81, the BT error is in the range of −20 K and 20K. This BT error has a relationship with the detector’s position. Due to the different position of each detector, the actual off-axis effects among the detectors are different. The BT errors, which are introduced by off-axis effects, change with the variation of the detectors’ positions. These conclusions can be proven through Figure 14a,b. Off-axis effects mainly cause the spectral shift. Therefore, the wavenumbers of the input spectrum and ideal spectrum have a deviation, and wavenumber deviations cause a large BT error. Figure 15c shows that the non-linearity response reduces the received energy in the range of 0.3 and 0.5 K, and the lost energy is regained through non-linearity correction in Figure 15d. The non-linearity response mainly affects the energy received by the detector. In the process of non-linearity correction, the initial correction parameter is given, and then the new estimated correction parameter is obtained using the low wavenumber out-of-band spectrum. The errors in this process reveal that the non-linearity response still saves a little after correction. This can be found in Figure 14f. Figure 14e gives the comparison before and after the off-axis effects. Section 4.3 introduces the off-axis correction, performed by the spectrum with the off-axis effects multiplying the SA^−1^ matrix. This operation can fulfill the off-axis correction effectively.

Figure 15 indicates the similar conclusions to Figure 14; thus, the detailed analyses are not given here. Two randomly selected detectors’ simulated results are used to fully prove the rationality and reliability of the whole simulation process.

## 5. Conclusions

The Michelson interferometer with fine spectral resolution is an important part in the Fourier spectrometer for the detection of atmosphere information. Understanding the instrument deeply will lead to better data processing and application, especially in the process of data calibration. In this paper, intensity simulation of the Michelson interferometer is introduced, which includes the off-axis effects and non-linearity response. The FY-4B GIIRS is taken as an example to illustrate the intensity simulation of the Michelson interferometer. Through this paper, how the intensity is obtained from a theoretical and practical perspective is clearly demonstrated.

This paper illustrates an intensity simulation process using the FY-4B GIIRS’s instrument parameters from a theoretical and practical perspective. Unlike polar orbiting satellites, geostationary satellites have more detectors in the focal plane, and every detector has its own ILS. These factors result in the complexity of simulations. Off-axis effects and the non-linearity response are the main factors in the simulation process. The ILS is defined as the shape change of the monochromatic light passing through the instrument to the focal plane. The results of ILS computation reveal the relationship between ILS and optical axis. Each detector has an independent ILS, which has a relationship with the detector’s site. Along with deviation from the optical, spectral stretching and the shifting of detectors are serious concerns. Following the above intensity simulation strategy, the intensity simulation results of some typical detectors are obtained to illustrate the variation of intensity for different detectors. The intensity is relatively abstract and is a direct result of the instrument. Fourier transform of the interferogram is carried out to obtain the spectrum and illustrate the influences of off-axis effect and the non-linearity response. In this paper, the off-axis effects and the non-linearity response are analyzed first and separately and then combined to be analyzed in the whole simulation process. Off-axis effects cause spectrum shift, and non-linearity responses reduce the energy received by the detector. These can affect the output intensity of the detector. The adding process of the off-axis effects and non-linearity response and the correction process of these two factors fully prove the whole simulation process to be correct.

The FY-4B geostationary satellite is planned for launch in 2020, and the GIIRS is a Fourier transform spectrometer and is a representative of square detector. Until this satellite is launched, the GIIRS’s simulation process can be used to obtain a better understanding of the instrument’s parameters and to develop methods for handling the possible problems of on-orbit testing. In future works, other factors will be considered, and a much more complete simulation system will be designed.

## Figures and Tables

**Table 1:** Explanation of parameters in the equations in this manuscript.

Parameters	Interpretation
I_total_	The total intensity (DC part + AC part)
I	The interference intensity
E_0_	Amplitude
X	Optical path difference
λ	Wavelength of the input light
σ	Actual wavenumber of the input light (σ = 1/λ)
σ_n_	Apparent wavenumber of the input light,
B	The intensity for DC part
R	Distance from the detector to the center of focal plane
f	Focal length
φ	The opening angle of the arc segmented by the detector
rc1, rc2, rmin, rmax	The distance between the detector and the optical axis
x_c_	Half height of the square detector
y_c_	Half width of the square detector
α_1_, α_2_	Opening angles of different arcs
Ω	The solid angle
θ	Off-axis angle
Spec	The measured spectrum
I	The ideal signal
m	The actual interference signal
$a_{2}$	The nonlinear parameter

## Appendix A

**Table A1:** ILS expressions of the square detector for off-axis positions.

$r_{c1}<r_{c2}$		
The Range of *r*	Corresponding wave-number range	ILS expression
$r_{\min}<r<r_{c1}$	σ1+rc12f2<σn<σ1+rmin2f2	$\frac{1}{4}-\frac{1}{2\pi }\arcsin(\frac{y_{c}-B}{r})-\frac{1}{2\pi }\arcsin(\frac{x_{c}-A}{r})$
$r_{c1}<r<r_{c2}$	σ1+rc22f2<σn<σ1+rc12f2	$\frac{1}{4}-\frac{1}{2\pi }\arcsin(\frac{y_{c}-B}{r})-\frac{1}{2\pi }\arccos(\frac{y_{c}+B}{r})$
$r_{c2}<r<r_{\max}$	σ1+rmax2f2<σn<σ1+rc22f2	$\frac{1}{4}-\frac{1}{2\pi }\arccos(\frac{y_{c}+B}{r})-\frac{1}{2\pi }\arccos(\frac{x_{c}+A}{r})$
$r_{c2}<r_{c1}$		
$r_{\min}<r<r_{c2}$	σ1+rc12f2<σn<σ1+rmin2f2	$\frac{1}{4}-\frac{1}{2\pi }\arcsin(\frac{y_{c}-B}{r})-\frac{1}{2\pi }\arcsin(\frac{y_{c}+B}{r})$
$r_{c2}<r<r_{c1}$	σ1+rc22f2<σn<σ1+rc12f2	$\frac{1}{4}-\frac{1}{2\pi }\arcsin(\frac{x_{c}-A}{r})-\frac{1}{2\pi }\arccos(\frac{x_{c}+A}{r})$
$r_{c1}<r<r_{\max}$	σ1+rmax2f2<σn<σ1+rc22f2	$\frac{1}{4}-\frac{1}{2\pi }\arccos(\frac{y_{c}+B}{r})-\frac{1}{2\pi }\arccos(\frac{x_{c}+A}{r})$
$r_{c2}=r_{c1}$		
$r_{\min}<r<r_{c2}$	σ1+rc22f2<σn<σ1+rmin2f2	$\frac{1}{4}-\frac{1}{2\pi }\arcsin(\frac{y_{c}-B}{r})-\frac{1}{2\pi }\arcsin(\frac{x_{c}-A}{r})$
$r_{c2}<r<r_{\max}$	σ1+rmax2f2<σn<σ1+rc22f2	$\frac{1}{4}-\frac{1}{2\pi }\arccos(\frac{y_{c}+B}{r})-\frac{1}{2\pi }\arccos(\frac{x_{c}+A}{r})$
